# Supplementary figures and images for: Temporal regulation of cold transcriptional response in switchgrass
Source: Front Plant Sci. 2022 Oct 10;13:998400. doi: 10.3389/fpls.2022.998400 (PMC9589291; doi:10.3389/fpls.2022.998400)

**A**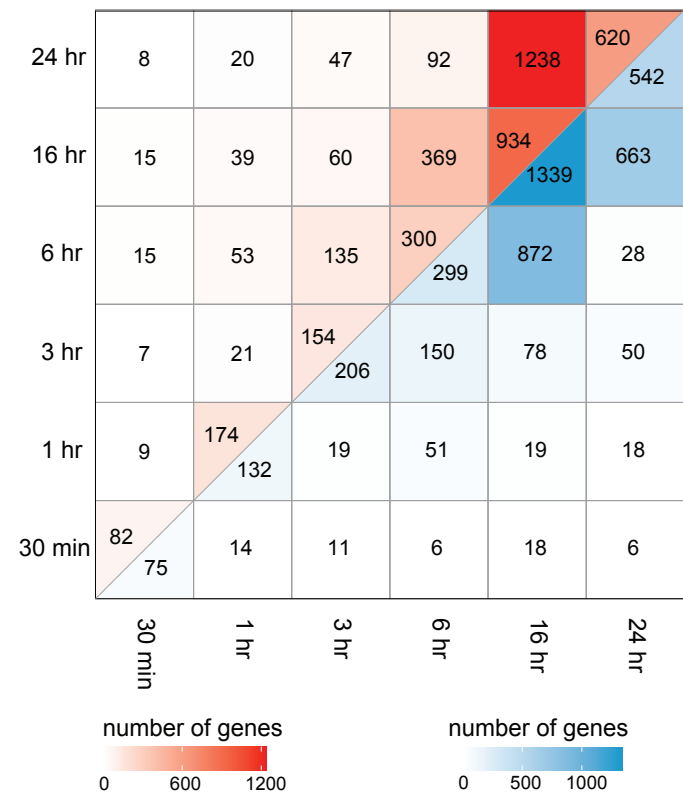**B**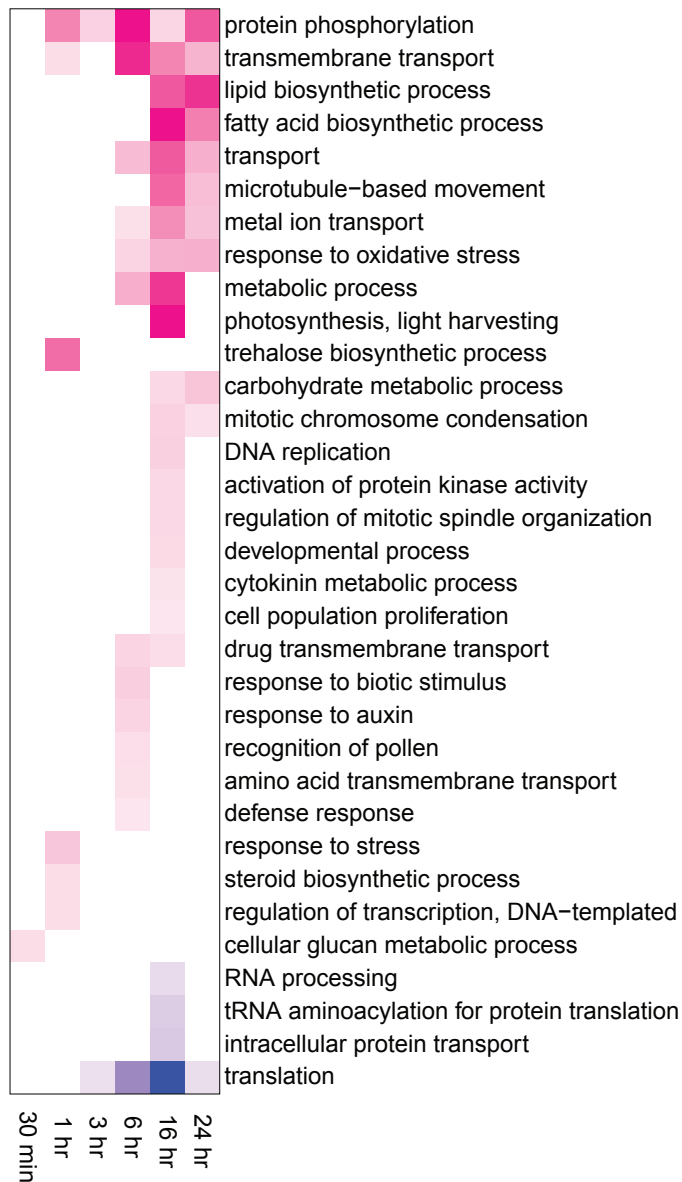**C**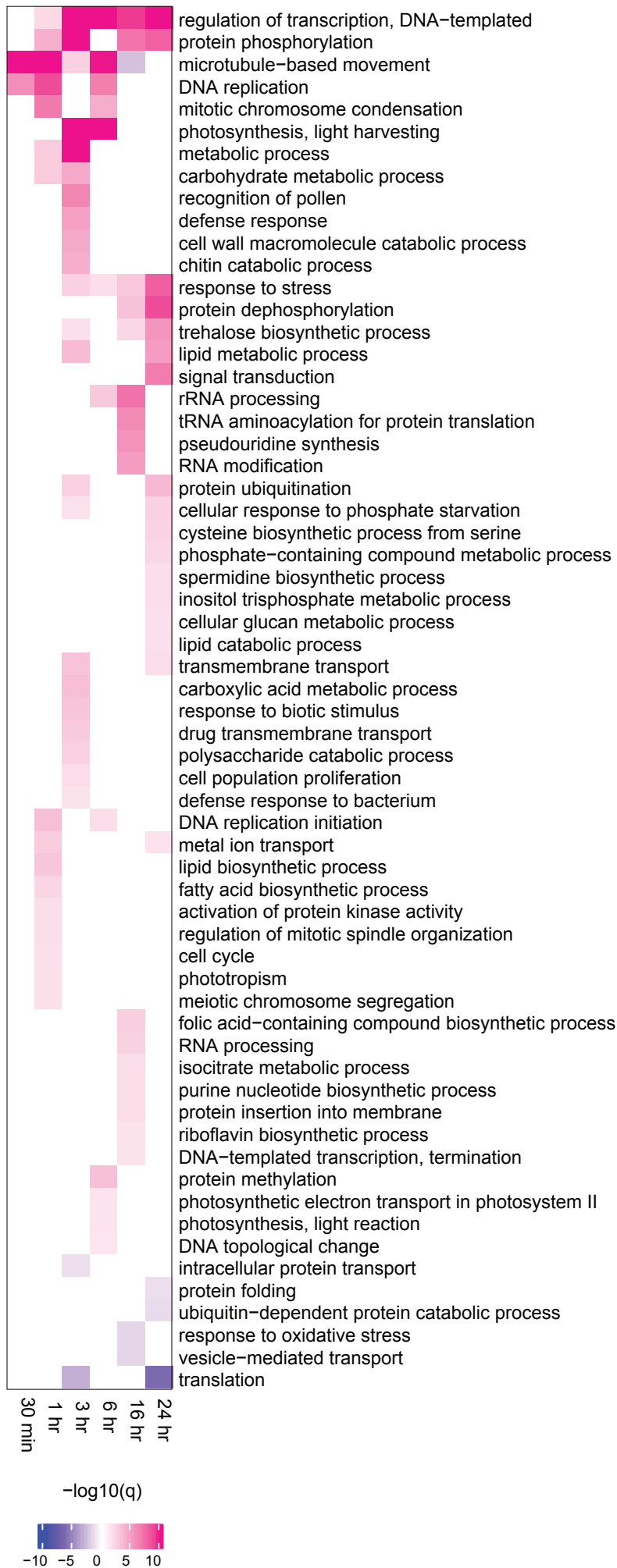

Supplement: Supplementary Figure 1 — Properties of cold-responsive genes at different time points. (A) Matrix showing the number of up-regulated (top left triangle) and down-regulated (bottom right triangle) genes at different time points after cold treatment. Color scale and number within the cell on the diagonal represent the count of time point-specific cold-responsive genes, while those in other cells indicate the number of responsive genes shared between two time points. For example, the number eight in the top left cell indicates that there are eight genes that are up-regulated at both 30 min and 24 hrs. (B, C) Biological process GO terms that are significantly enriched (q ≤ 0.05) for genes that are down-regulated (B) or up-regulated (C) at different time points. Color scale: -log10(q) for over-representative GO terms, and log10(q) for under-representative GO terms. [file Image_1.pdf]

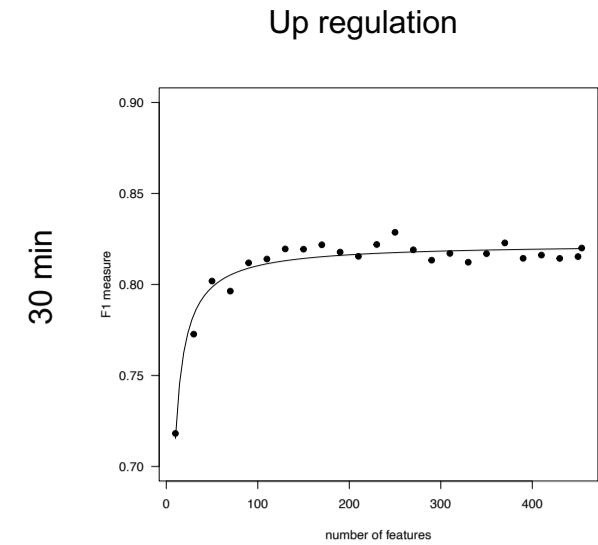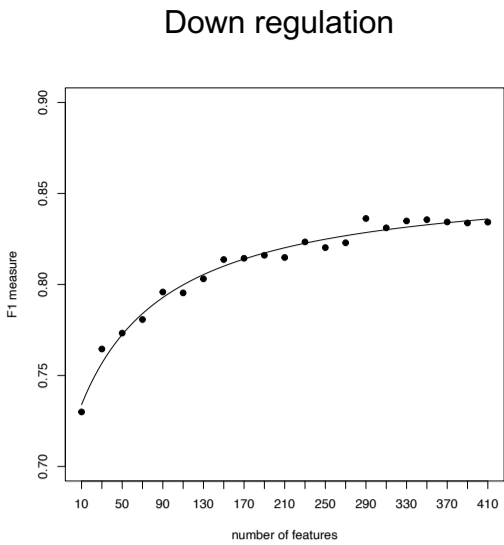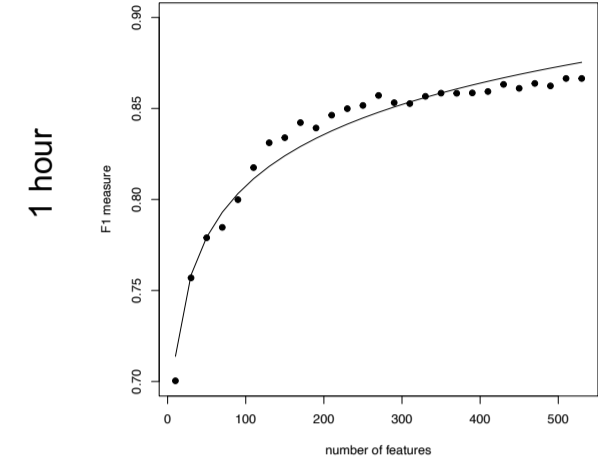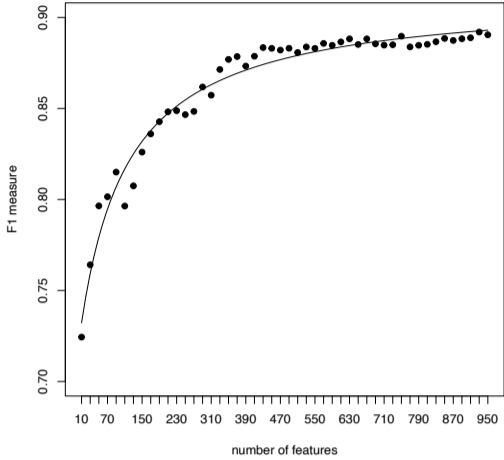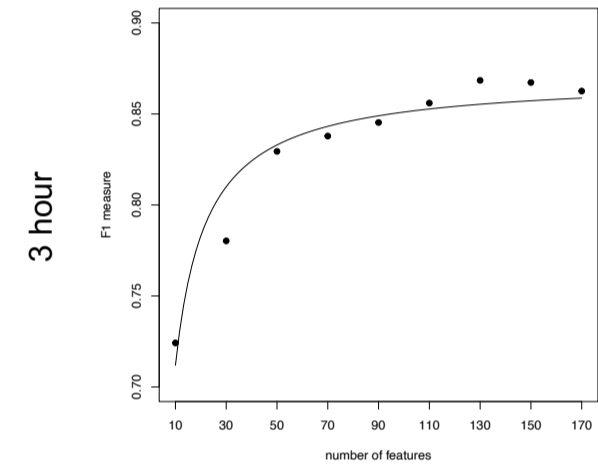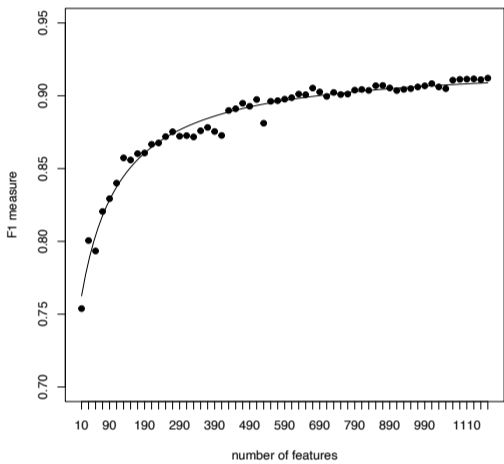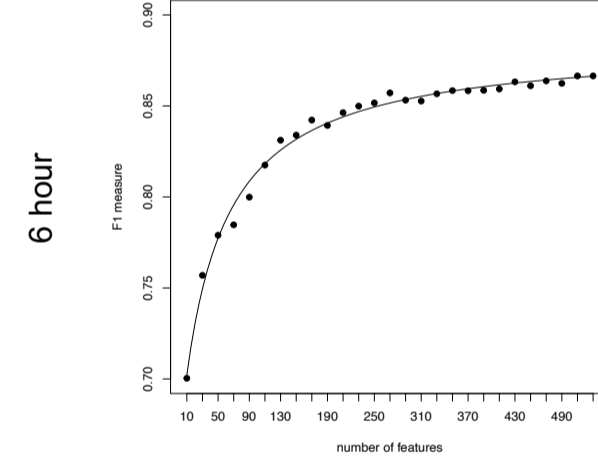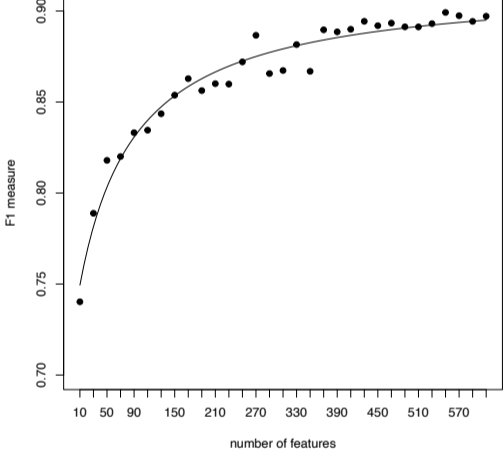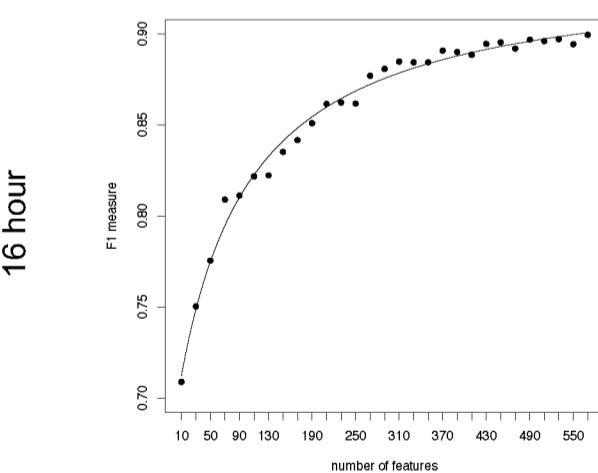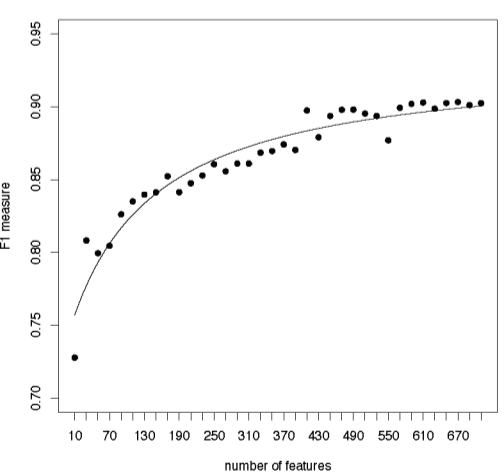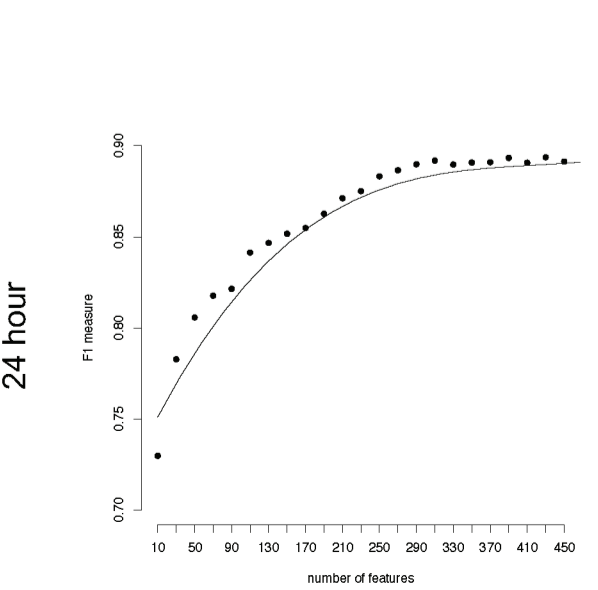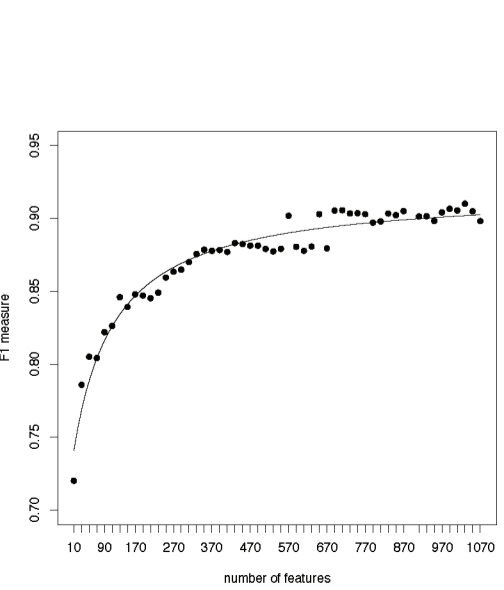

Supplement: Supplementary Figure 2 — Feature selection. Graphs show the relationship between the F1score and the number of features in time point models distinguishing genes up-regulated (left panel) or down-regulated (right panel) after cold treatment from non-responsive genes. The data points were fitted using the Michaelis-Menten Equation. [file Image_2.pdf]

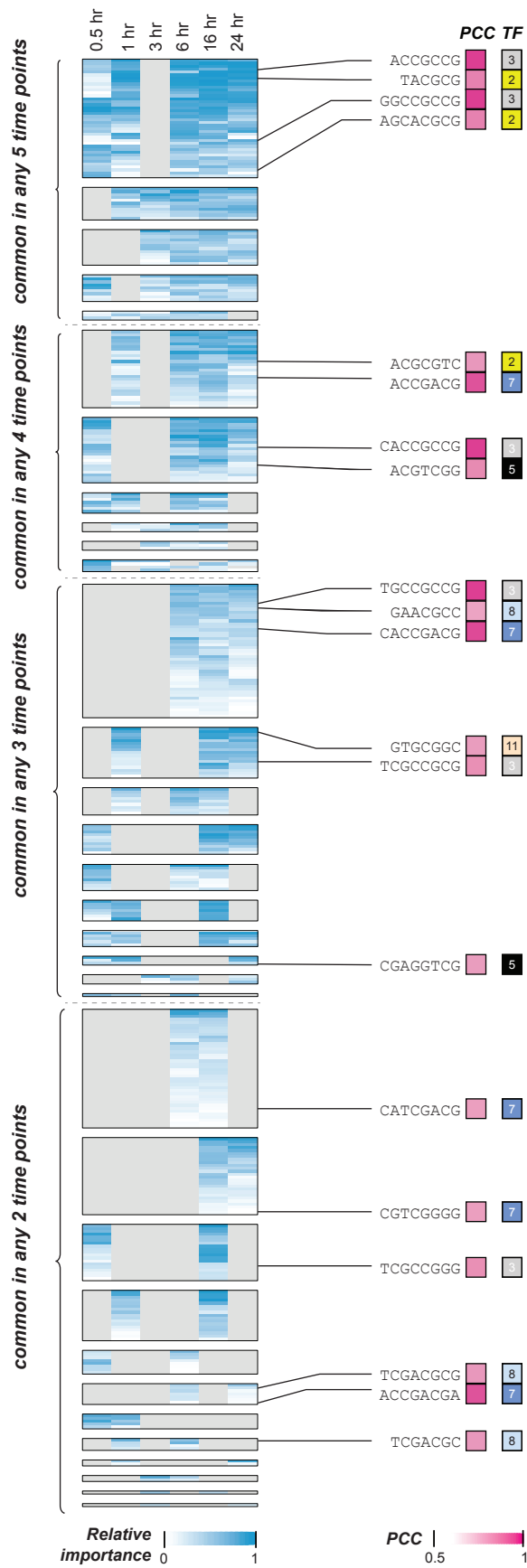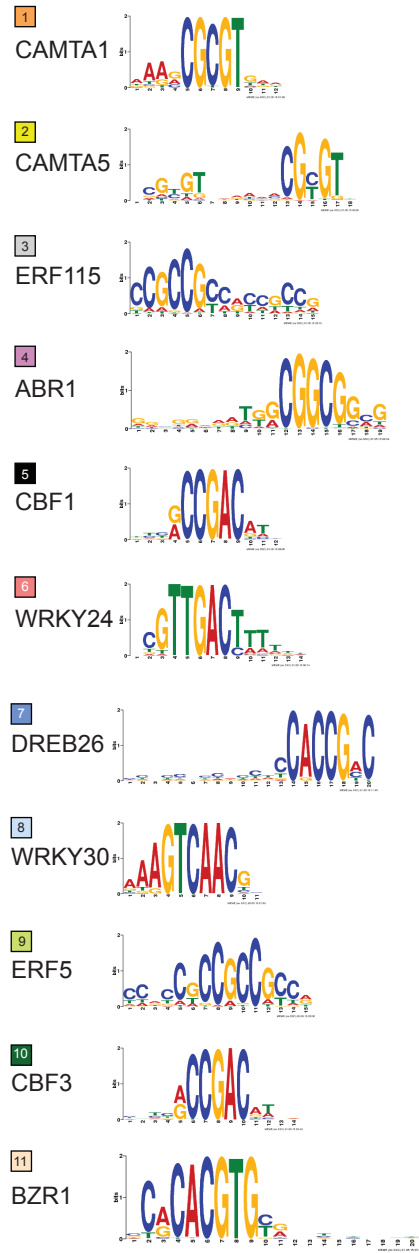

Supplement: Supplementary Figure 3 — pCREs that were identified from models predictive of genes up-regulated in >1 time points and their resemblances with known cold-CREs. [file Image_3.pdf]

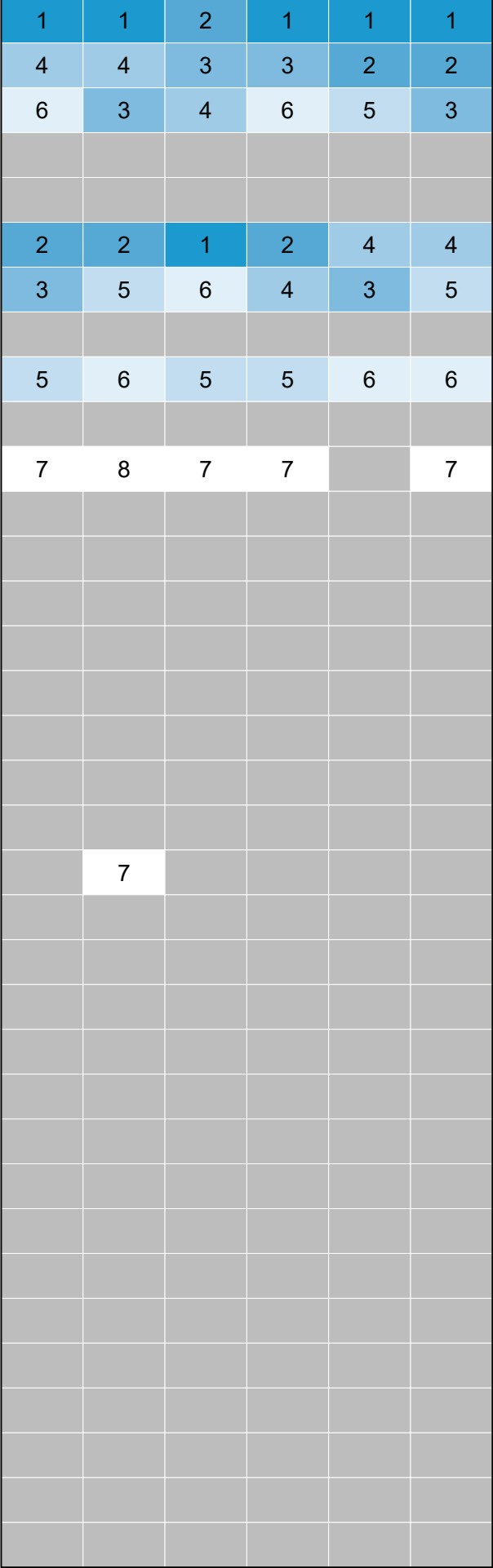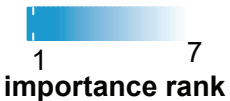

Supplement: Supplementary Figure 4 — Heatmap showing feature importance in the cold-TFBM models. Color scale and numbers in the cells represent the importance rank of features that have positive Gini indexes, the darker color and smaller number, the more important a feature was. Gray color indicates that the Gini index for the feature was negative. [file Image_4.pdf]

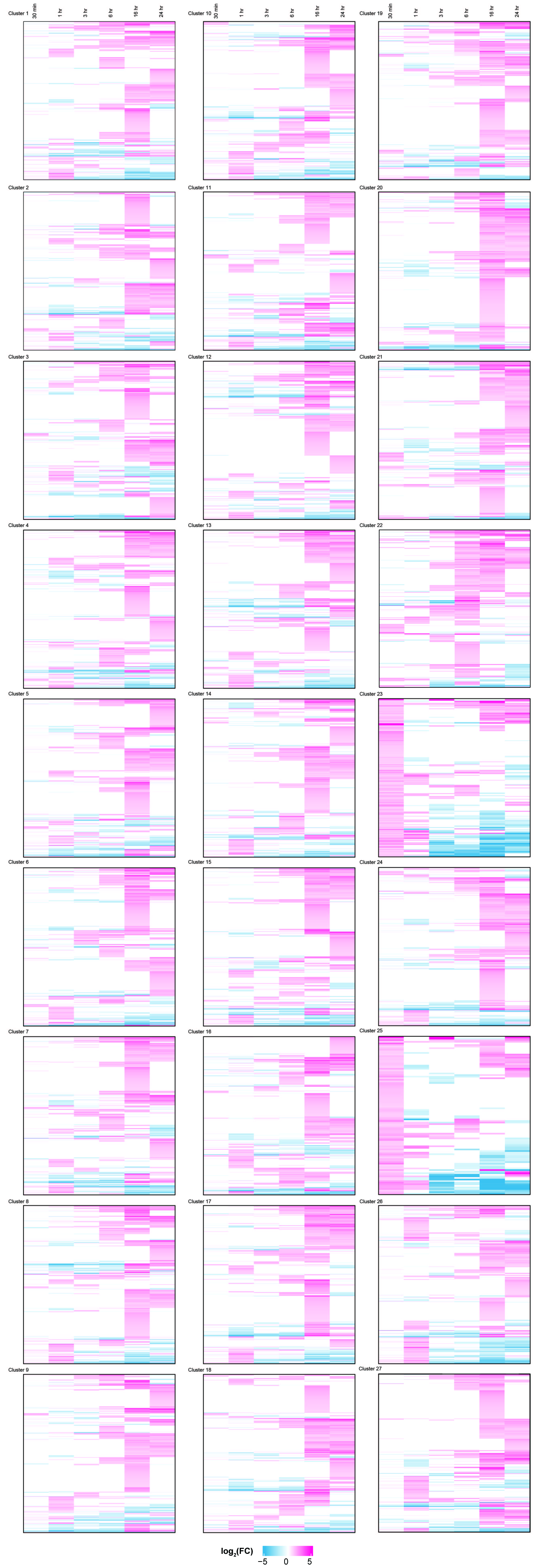

Supplement: Supplementary Figure 6 — Heatmaps showing the degrees of differential expression of genes that contain pCREs from different pCRE clusters at different time points after cold treatment. Color scale indicates log fold change values. [file Image_6.tif]

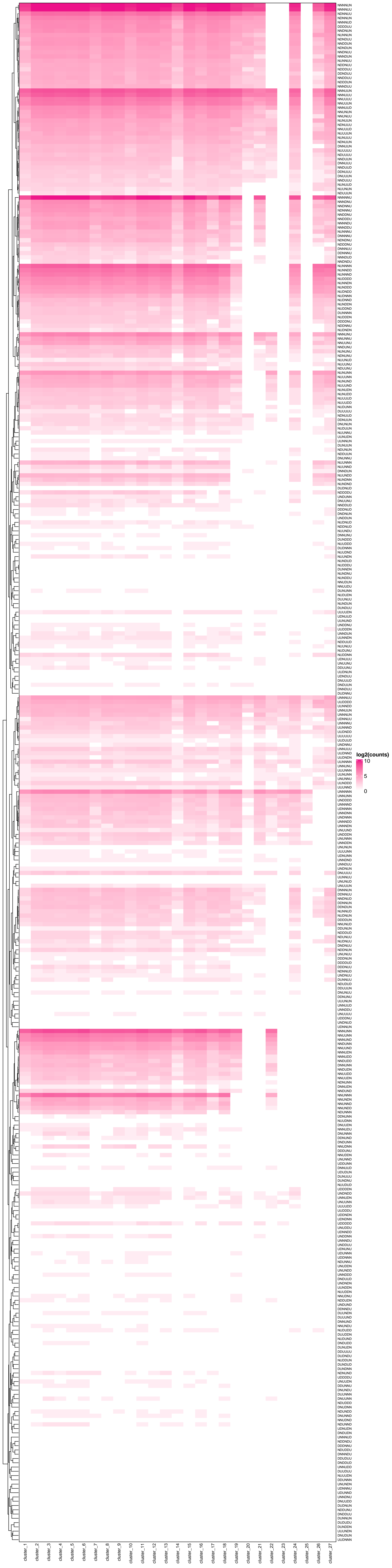

Supplement: Supplementary Figure 7 — Frequency of genes showing a specific expression profiles (e.g., NNUDNN, y-axis) and containing pCREs that belong to different pCRE clusters (x-axis). Color scale indicates log2(counts) of genes showing the expression profile. U, up-regulated; D, down-regulated; N, non-responsive. [file Image_7.pdf]

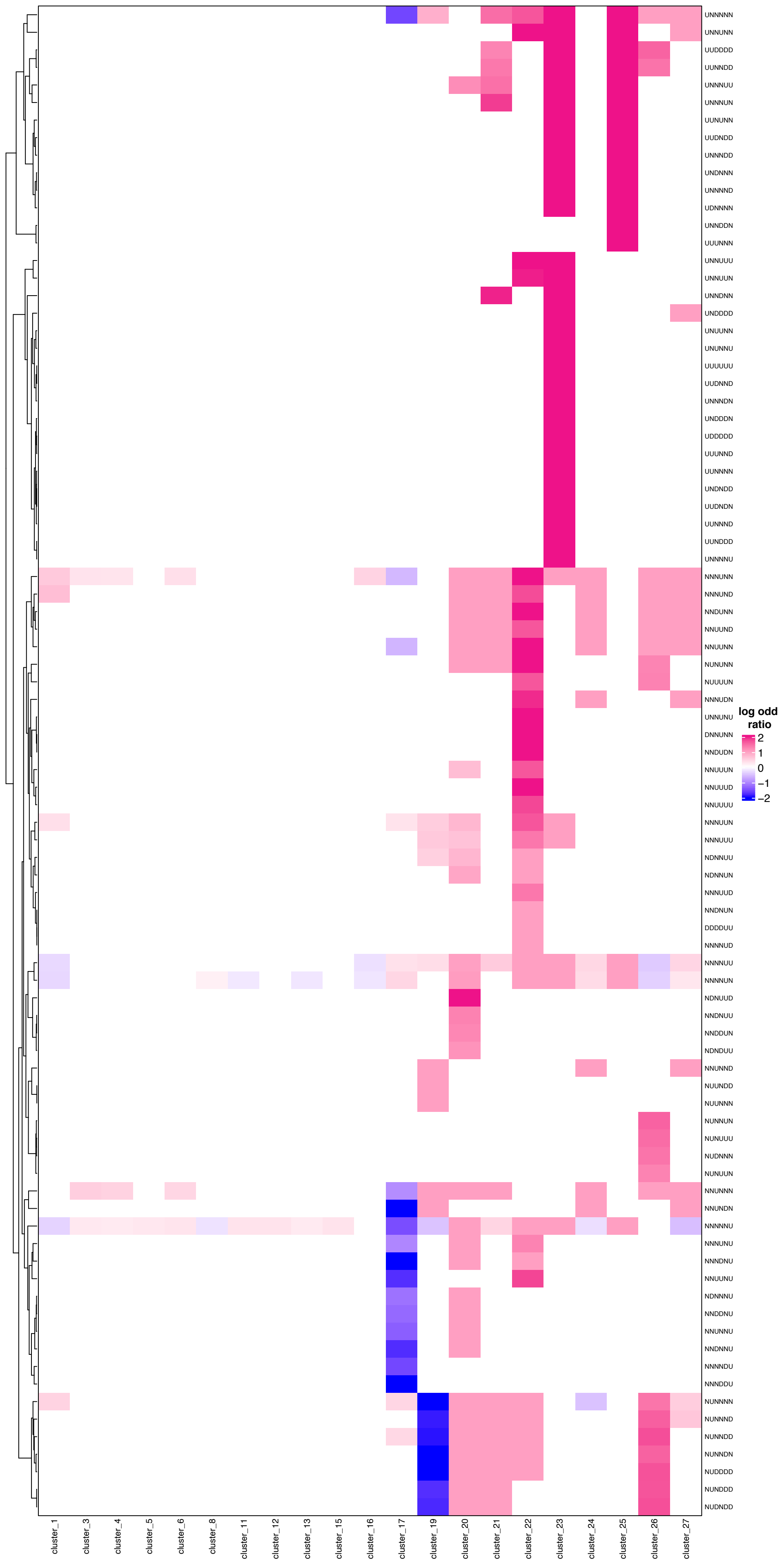

Supplement: Supplementary Figure 8 — Degrees of enrichment of genes containing pCREs from a pCRE cluster (x-axis) that also have a specific expression profile (e.g., NNUDNN, y-axis). The color scale represents the log odds ratio, which was calculated as ratios between two values. The first value is the number of genes containing pCRE in cluster C and having expression profile P divided by the number of genes with C and not in P. The second value is the number of genes with no C but in P divided by the number of genes without C and not in P. [file Image_8.pdf]
