# Supplementary material for: Temporal regulation of cold transcriptional response in switchgrass
Source: Front Plant Sci. 2022 Oct 10;13:998400. doi: 10.3389/fpls.2022.998400 (PMC9589291; doi:10.3389/fpls.2022.998400)
Supplement: Supplementary Figure 5 — A dendrogram showing relationships among general and time point-specific pCREs based on pairwise PCC distances. The dendrogram is clustered based on the similarity threshold of 0.39. [file Image_5.pdf]

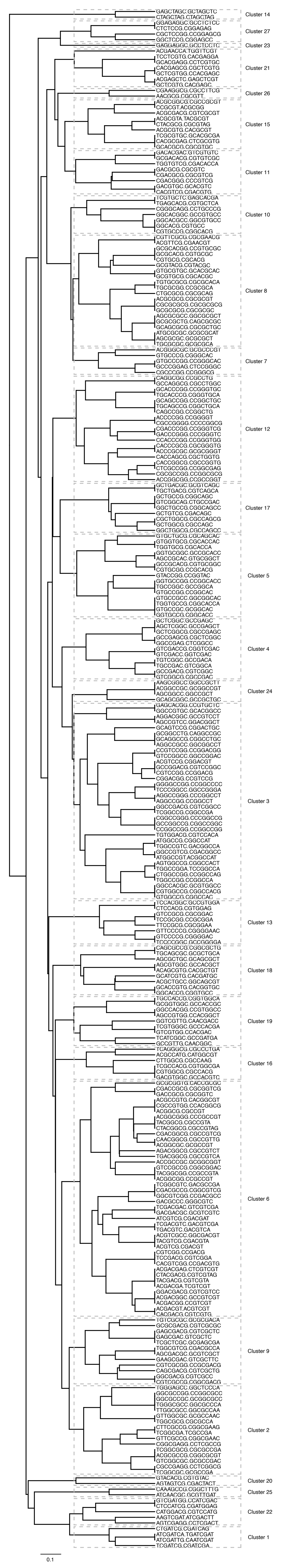
Cluster 14
Cluster 27
Cluster 23
Cluster 21
Cluster 26
Cluster 15
Cluster 11
Cluster 10
Cluster 8
Cluster 7
Cluster 12
Cluster 17
Cluster 5
Cluster 4
Cluster 24
Cluster 3
Cluster 13
Cluster 18
Cluster 19
Cluster 16
Cluster 6
Cluster 9
Cluster 2
Cluster 20
Cluster 25
Cluster 22
Cluster 1
0.1
